# Hospital utilization in Indonesia in 2018: do urban–rural disparities exist?

**DOI:** 10.1186/s12913-022-07896-5

**Published:** 2022-04-12

**Authors:** Ratna Dwi Wulandari, Agung Dwi Laksono, Zainul Khaqiqi Nantabah, Nikmatur Rohmah, Zuardin Zuardin

**Affiliations:** 1 Faculty of Public Health, Universitas Airlangga, Surabaya, Indonesia; 2 The Airlangga Centre for Health Policy (ACeHAP), Surabaya, Indonesia; 3 National Research and Innovation Agency, Republic of Indonesia, Jakarta, Indonesia; 4 Faculty of Health Science, Muhammadiyah University of Jember, East Java, Indonesia; 5 Faculty of Psychology and Health, UIN Sunan Ampel, Surabaya, Indonesia

**Keywords:** Hospital utilization, Healthcare evaluation, Healthcare access, Public health

## Abstract

**Background:**

The government must ensure equality in health services access, minimizing existing disparities between urban and rural areas. The referral system in Indonesia is conceptually sound. However, there are still problems of uneven service access, and there is an accumulation of patients in certain hospitals. The study aims to analyze the urban–rural disparities in hospital utilization in Indonesia.

**Methods:**

The study used secondary data from the 2018 Indonesian Basic Health Survey. This cross-sectional study gathered 629,370 respondents through stratification and multistage random sampling. In addition to the kind of home and hospital utilization, the study looked at age, gender, marital status, education, occupation, wealth, and health insurance as control factors. The research employed multinomial logistic regression to evaluate the data in the final step.

**Results:**

According to the findings, someone who lives in an urban region has 1.493 times higher odds of using outpatient hospital services than someone in a rural area (AOR 1.493; 95% CI 1.489–1.498). Meanwhile, someone who lives in an urban region has 1.075 times higher odds of using an inpatient facility hospital than someone who lives in a rural one (AOR 1.075; 95% CI 1.073–1.077). Furthermore, someone living in an urban region has 1.208 times higher odds than someone who lives in a rural area using outpatient and inpatient hospital services simultaneously (AOR 1.208; 95% CI 1.204–1.212).

**Conclusion:**

The study concluded there were urban–rural disparities in hospital utilization in Indonesia.

## Background

The World Health Organization identifies six principal components (building blocks) in the health system framework. One of these components is health services [1]. For the referral process to function appropriately in health services, it is necessary to have a system that regulates the transfer of patients from one place to another [2]. In Indonesia, the referral process adheres to a tiered health service system: the first or primary level of service, the second or secondary level, and the third or tertiary level [3]. In the referral system in Indonesia, the hospital is an advanced level referral health facility and the gateway to the highest health facility. The situation means that the government expected hospitals to help and solve community problems related to health/medical entirely. Even though the concept is good, there are still problems of uneven service access and accumulation of patients in certain hospitals as the last level in the referral system [4].

In Indonesia, around 110 regional referral hospitals, 20 provincial referral hospitals, and 14 central referral hospitals [5]. Although the government has developed a regional referral system, there are still challenges in geography, health care facilities availability, and patients. Geographical conditions in archipelagic Indonesia have proven to cause disparities between regions. Access to services is a significant obstacle, which has implications for the uneven distribution of health care facilities [6, 7].

From the patient side, although health costs for some people are no longer a problem because of National Health Insurance (NHI), the biggest obstacle is the cost of transportation to reach services [8, 9]. In addition, the Indonesian people still adhere to a solid cultural and kinship system so that it dramatically influences the decision-making to use health services [10]. Meanwhile, there are challenges with human resources issues, the availability of infrastructure and medical equipment, and drugs [4]. The government has developed a program to accelerate service access to overcome these problems, especially in remote, underdeveloped, border, and island areas. Another policy form is releasing the *Nusantara Sehat* (healthy archipelago) program. This program explicitly places health workers in remote, underdeveloped, border, and island areas [11].

Referring to the global commitments contained in the Sustainable Development Goals (SDGs), Indonesia must meet the targets agreed in the SDGs. This issue is related to the 3rd goal in the health sector, namely a healthy and prosperous life associated with Universal Health Coverage (UHC). Various other SDGs goals also listed the achievement of health indicators. It is necessary to have government support and commitment to prepare and provide adequate and sufficient infrastructure and health resources for the entire community so that there is no disparity in health services throughout Indonesia to achieve the SDG’s target [12].

Several studies widely documented disparities in health services in studies in various countries. The difference generally refers to the health status and services between populations in an area [13–15]. We can view the gap from multiple dimensions, including social class, economy, age, education, geography, language, gender, persons with disabilities, citizenship, gender, and sexual orientation [16, 17]. The problem is, in Indonesia, even though the lower economic community is the group that most needs health services, access to health services is still concentrated in the upper financial community [18, 19].

Previous studies found that disparities in access to health care facilities can also exist in Iran and China. Some areas have very developed health services but are less advanced [20, 21]. One previous study in Indonesia that analyzed healthcare utilization among children under five found that children living in rural areas and from low-income families tended to choose primary health centers [22]. On the other hand, another study in Taiwan informed that Universal Health Coverage could minimize psychiatric services inequality in urban and rural areas [23]. Moreover, in Indonesia, NHI positively affects service utilization in all health care facilities, both government and private [24–26].

Based on the explanation of previous studies, several existing disparities in hospital utilization are urban–rural, age, socioeconomic, geography, insurance ownership, language, gender, persons with disabilities, citizenship, gender, and sexual orientation. Based on the research background, the study aims to analyze the urban–rural disparities in hospital utilization in Indonesia. The study included other relevant variables as controls.

## Materials and methods

### Data source

The research employed secondary data from the 2018 Indonesian Basic Health Survey. Meanwhile, the study was a national-scale cross-sectional survey undertaken by the Republic of Indonesia’s Ministry of Health. The survey collected data during May–July 2018 through interviews with Household Instruments and Individual Instruments.

The 2018 Indonesian Basic Health Survey population is all households in Indonesia. The survey uses the 2018 National Socio-Economic Survey sample framework, conducted in March 2018. Moreover, the survey visited the target sample of 300,000 households from 30,000 of the 2018 Socio-Economic Survey census blocks (run by the Central Statistics Agency) [27].

The survey uses the PPS (probability proportional to size) method using systematic linear sampling, with Two-Stage Sampling: Stage 1: Implicit stratification of all census blocks resulting from the 2010 Population Census based on welfare strata. The sample survey selected by PPS to be the sampling frame for the selection of census blocks from the master frame of 720,000 Census Blocks from the 2010 Population Census, 180,000 Census Blocks (25%). The survey determined several census blocks with the PPS method in each urban/rural strata per regency/city to produce a Census Block Sample List. The total number of selected Census Blocks is 30,000 Census Blocks. Stage 2: Selecting ten households in each Census Block updated by systematic sampling with the highest implicit stratification of education completed by the Head of the Household to maintain the representation of the diversity value of household characteristics. Individuals sampled in the 2018 Indonesian Basic Health Survey to be interviewed all household members in the selected household [27].

The population in this study was all adults (≥ 15 years old) in Indonesia. The study described 629,370 respondents as a weighted sample based on the sampling methods.

### Outcome variable

The study used hospital utilization as the outcome variable—adults’ access to hospitals, whether outpatient or inpatient, was hospital utilization. The hospital utilization consists of four categories: unutilized, outpatient, inpatient, and outpatient as well as an inpatient. On the other hand, outpatient hospitalizations were restricted to the previous month, whereas the study determined inpatient hospitalizations to the past year. The poll requested respondents to recollect outpatient and inpatient incidents correctly [27].

### Exposure variable

The analysis employed the type of residence as an exposure variable in the study. The survey classified the type of residence given into two categories: urban and rural. Furthermore, the study used the Indonesian Central Statistics Agency’s provisions for urban–rural categorization in the survey.

### Control variables

The study used seven elements as control variables as part of those variables. The seven criteria were age, gender, marital status, education level, work type, wealth status, and health insurance ownership.

The study determined the age based on the last birthday that the respondent passed. Gender, on the other hand, was divided into two categories in the survey: male and female. The study also classified marital status into three groups: never in a union, married/living with a partner, and divorced/widowed.

The respondent’s education is their acknowledgment of their most recent diploma. There are four levels of education in the study: no education, primary, secondary, and higher education. Meanwhile, the work typically consists of six types: no work, civil servant/army/police, private sector, entrepreneur, farmer/fisherman/labor, others.

The survey used the wealth index formula to identify wealth status in the study. The survey calculated the wealth index using a weighted average of a family’s total spending. Meanwhile, the survey computed the wealth index using primary household expenditures such as health insurance, food, and lodging, among other things. Furthermore, the poll divided the income index into five categories: the poorest, poorer, middle, richer, and the richest [19]. Moreover, the survey splits health insurance ownership into four types: uninsured, government-run insurance, private-run insurance, and have government-run and private-run insurance.

### Data analysis

First, the study utilized the Chi-Square test to produce a bivariate comparison for the dichotomous variable. At the same time, the study used the T-test for the continuous variable (age). Furthermore, a collinearity test was utilized in the study to ensure that the independent variables in the final regression model did not have a strong connection. The analysis used a multinomial logistic regression in the study’s last point. The study utilized this previous test to investigate the multivariate relationship between all independent variables and hospital utilization in the survey. The research used the IBM SPSS 22 application throughout the statistical analysis phase in the investigation.

In contrast, the study used ArcGIS 10.3 (ESRI Inc., Redlands, CA, USA) to map hospital utilization among the elderly in Indonesia by the province in 2018. The Indonesian Bureau of Statistics submitted a shapefile of administrative border polygons for the analysis.

## Results

**Fig. 1:**
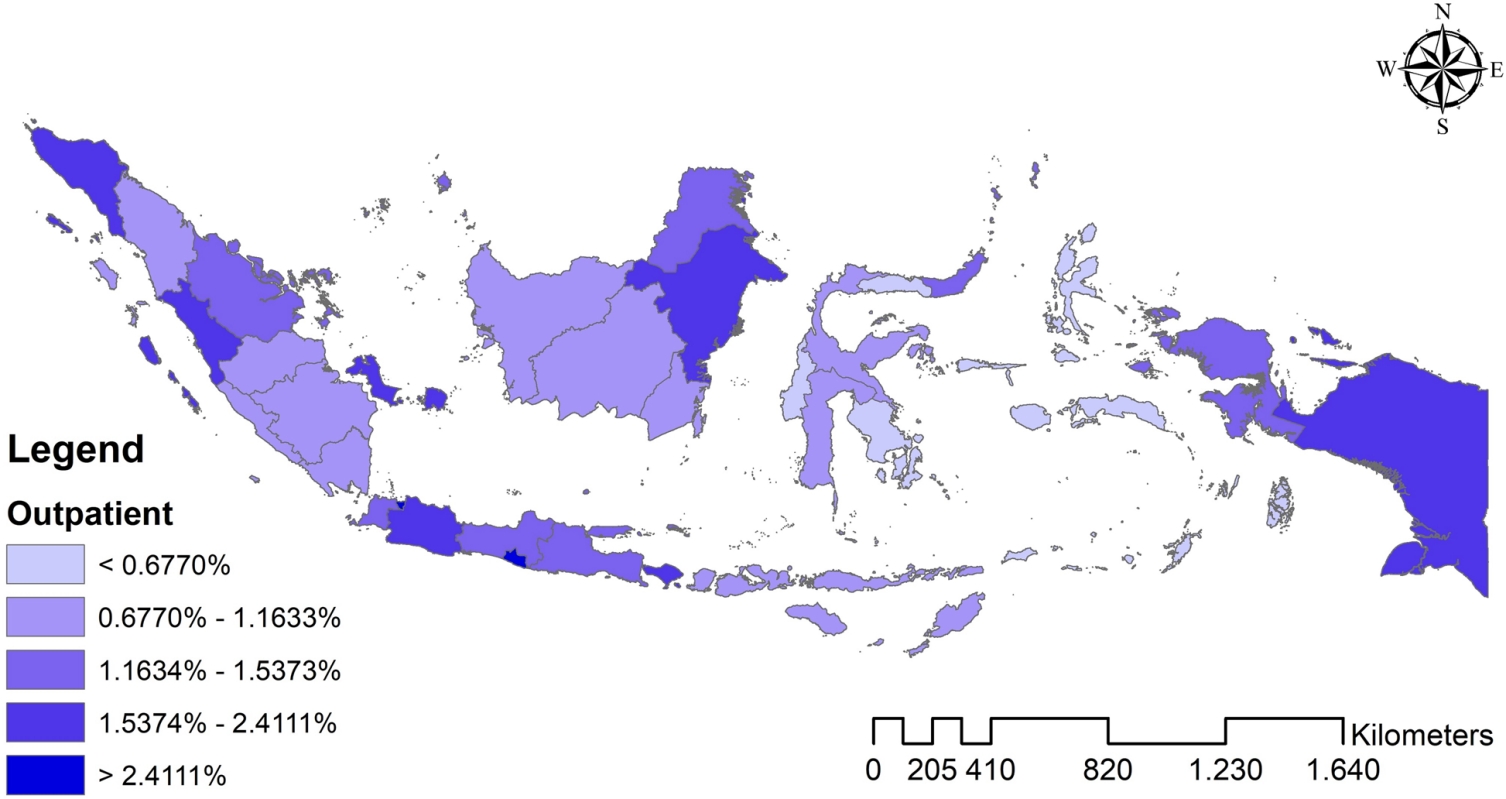
Distribution map of outpatient by the province in Indonesia in 2018

**Fig. 2:**
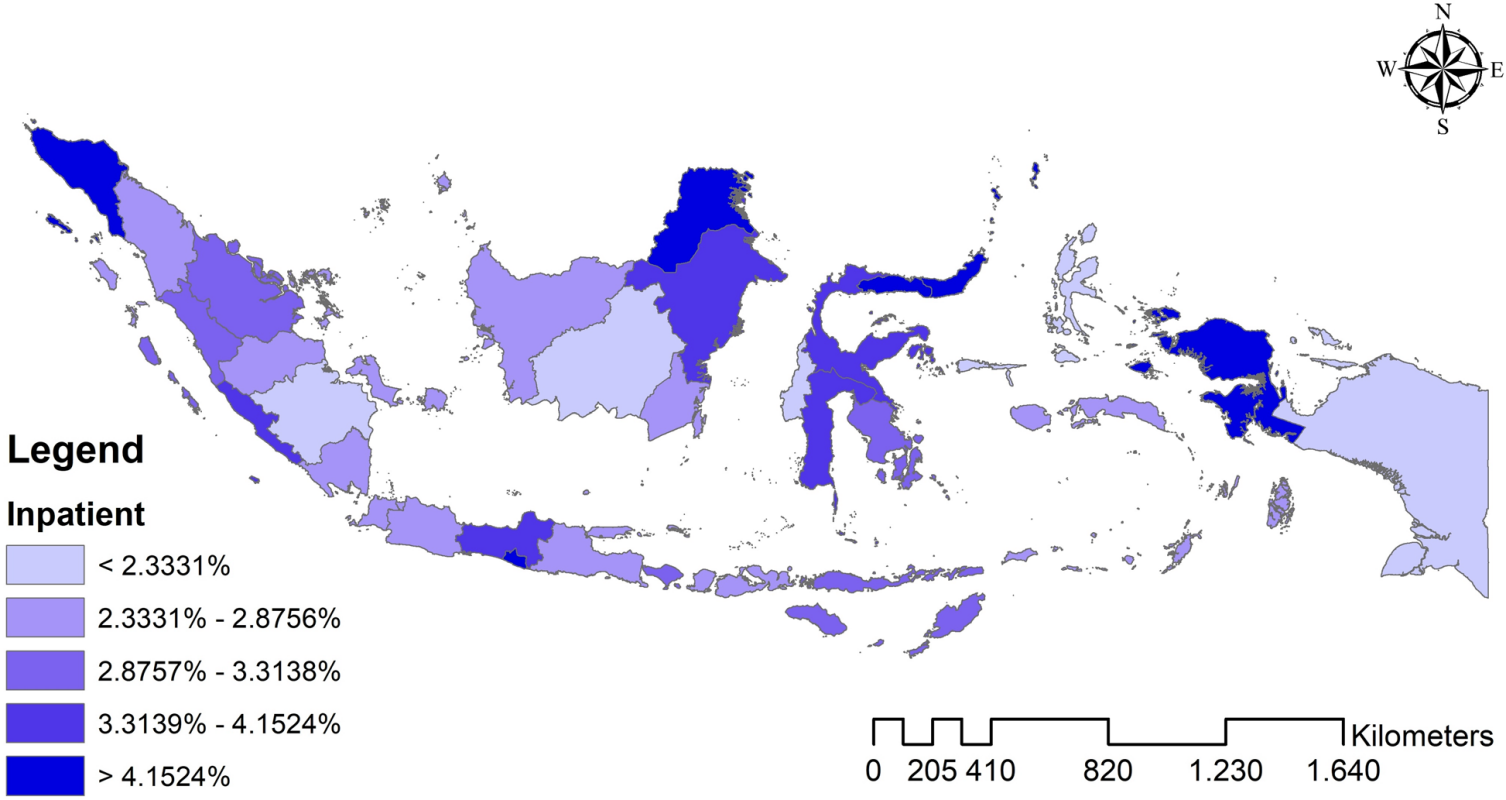
Distribution map of inpatient by the province in Indonesia in 2018

**Fig. 3:**
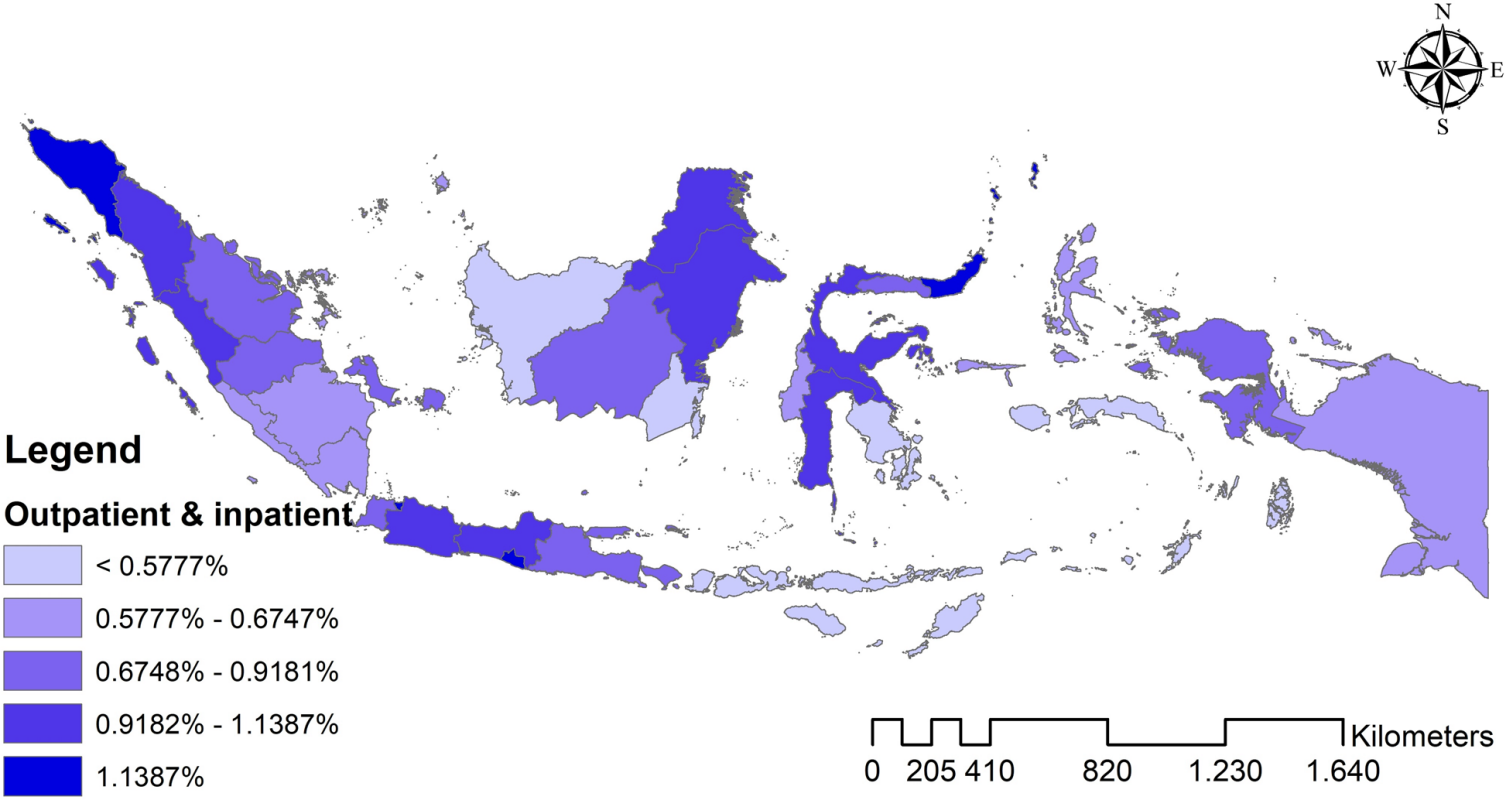
Distribution map of outpatient as well as an inpatient by the province in Indonesia in 2018

The analysis results found that Indonesia’s national average hospital utilization in 2018 was outpatient 1.465%, inpatient 3.053%, and outpatient-inpatient simultaneous 0.934%. Meanwhile, Fig. 1–3 shows the distribution map of hospital utilization by the province in Indonesia in 2018. Figure 1 shows the distribution map of outpatient; Fig. 2 shows the distribution map of inpatient; Fig. 3 shows the distribution map of outpatient-inpatient simultaneous. The three maps indicate no particular trend pattern spatially; the distribution of hospital utilization proportion looks random.

Table 1 shows descriptive statistics of the respondents. Unutilized people are mainly in urban and rural areas based on hospital utilization. Meanwhile, those who live in rural areas have a slightly older average age than those in urban areas. Moreover, based on gender, females lead in both urban and rural areas.

**Table 1:** Descriptive statistic of respondents (*n* = 629,370)

Elderly Characteristics	Type of Residence		*p*-value
	**Urban** **(*n* = 271,814)**	**Rural** **(*n* = 357,556)**	
**Hospital utilization**			< 0.001
Unutilized	93.5%	95.8%	
Outpatient	1.9%	0.9%	
Inpatient	3.4%	2.6%	
Outpatient and inpatient simultaneous	1.1%	0.7%	
**Age (mean)**	(38.89)	(39.9)	< 0.001
**Gender**			
Male	49.8%	49.8%	
Female	50.2%	50.2%	
**Marital status**			< 0.001
Never in union	25.3%	20.1%	
Married/Living with a partner	66.5%	71.4%	
Divorced/Widowed	8.2%	8.5%	
**Education level**			< 0.001
No education	3.7%	8.3%	
Primary	48.6%	68.8%	
Secondary	36.0%	18.7%	
Higher	11.7%	4.2%	
**Work type**			< 0.001
No work	39.8%	34.7%	
Civil servant/army/police	3.8%	1.8%	
Private sector	14.6%	4.6%	
Entrepreneur	17.3%	10.6%	
Farmer/fisherman/labor	18.7%	43.0%	
Others	5.8%	5.2%	
**Wealth status**			< 0.001
Poorest	12.2%	23.5%	
Poorer	16.0%	21.6%	
Middle	18.1%	20.8%	
Richer	19.9%	21.5%	
Richest	33.8%	12.7%	
**Health Insurance**			< 0.001
Uninsured	29.1%	37.1%	
Government-run insurance	64.5%	61.3%	
Private-run insurance	4.9%	1.2%	
Government-run and Private-run insurance	1.6%	0.4%	

Table 1 shows those married or living with a partner lead in urban and rural areas. On the other hand, secondary education represents both urban and rural areas. After this, based on work type, those who do not work led in the urban area, and meanwhile, farmer/fisherman/labor led in the rural area.

According to wealth status, the richest heald in the urban area. Contrary, the poorest ruled in rural areas. Then, based on health insurance ownership, those who have government-run insurance are in urban and rural areas.

Table 2 shows the results of the collinearity test of hospital utilization in Indonesia. The analysis results demonstrate no strong association between the independent variables.

**Table 2:** Results for the collinearity test of hospital utilization in Indonesia in 2018 (*n* = 629,370)

Variables	Collinearity Statistics	
	**Tolerance**	**VIF**
Type of residence	0.889	1.125
Age	0.552	1.813
Gender	0.828	1.208
Marital status	0.544	1.837
Education level	0.794	1.260
Work type	0.827	1.209
Wealth status	0.851	1.175
Health Insurance	0.959	1.042

^*^Dependent Variable: Hospital utilization

Table 2 demonstrates that the tolerance value for all variables is more significant than 0.10. For all factors, the variance inflation factor (VIF) value is less than 10.00. The study then noted that the regression model exhibited no signs of multicollinearity, indicating the test’s decision-making foundation.

Table 3 shows the result of multinomial logistic regression of hospital utilization in Indonesia. The analysis in this final stage uses ‘hospital unutilized’ as a reference.

**Table 3:** The result of multinomial logistic regression of hospital utilization in Indonesia in 2018 (*n* = 629,370)

Predictor	Outpatient			Inpatient			Outpatient and inpatient simultaneous		
	**AOR**	**95% CI**		**AOR**	**95% CI**		**AOR**	**95% CI**	
		**LB**	**UB**		**LB**	**UB**		**LB**	**UB**
Residence: Urban	*1.493	1.489	1.498	*1.075	1.073	1.077	*1.208	1.204	1.212
Residence: Rural	-	-	-	-	-	-	-	-	-
Age	*1.046	1.046	1.046	*1.004	1.004	1.004	*1.041	1.040	1.041
Gender: Male	*0.926	0.923	0.929	*0.674	0.673	0.675	*1.115	1.111	1.119
Gender: Female	-	-	-	-	-	-	-	-	-
Marital: Never in union	*1.462	1.453	1.471	*0.487	0.485	0.490	*0.980	0.972	0.988
Marital: Married/Living with partner	*1.268	1.264	1.273	*1.221	1.217	1.225	*1.415	1.408	1.422
Marital: Divorced/Widowed	-	-	-	-	-	-	-	-	-
Education: No Education	*0.505	0.502	0.509	*0.802	0.798	0.806	*0.922	0.914	0.930
Education: Primary	*0.864	0.860	0.868	*0.824	0.822	0.827	*1.220	1.212	1.227
Education: Secondary	*0.966	0.962	0.970	*0.892	0.890	0.895	*1.113	1.106	1.119
Education: Higher	-	-	-	-	-	-	-	-	-
Work: no work	*1.195	1.189	1.201	*1.124	1.120	1.128	*1.704	1.693	1.715
Work: civil servant/army/police	*0.907	0.901	0.914	*0.672	0.668	0.675	*0.833	0.825	0.841
Work: private sector	*0.763	0.758	0.768	*0.746	0.743	0.749	*0.696	0.690	0.702
Work: entrepreneur	*0.842	0.837	0.847	*0.749	0.746	0.752	*0.903	0.896	0.910
Work: farmer/fisherman/labor	*0.668	0.664	0.672	*0.659	0.657	0.662	*0.736	0.731	0.742
Work: others	-	-	-	-	-	-	-	-	-
Wealth: Poorest	*0.388	0.387	0.390	*0.489	0.488	0.491	*0.253	0.251	0.254
Wealth: Poorer	*0.447	0.445	0.449	*0.597	0.595	0.599	*0.389	0.387	0.391
Wealth: Middle	*0.586	0.584	0.588	*0.688	0.686	0.689	*0.500	0.498	0.503
Wealth: Richer	*0.689	0.687	0.691	*0.823	0.821	0.825	*0.658	0.655	0.661
Wealth: Richest	-	-	-	-	-	-	-	-	-
Insurance: Uninsured	*0.168	0.167	0.170	*0.231	0.230	0.233	*0.125	0.123	0.126
Insurance: Government-run	*0.452	0.448	0.455	*0.638	0.634	0.642	*0.543	0.538	0.549
Insurance: Private-run	*0.540	0.535	0.544	*0.598	0.594	0.602	*0.581	0.575	0.588
Insurance: Government-run & Private-run	-	-	-	-	-	-	-	-	-

∗ *p* < 0.010; ∗  ∗ *p* < 0.001, *AOR* Adjusted Odds Ratio, *CI* confidence interval, *LB* lower bound, *LB* lower bound

Table 3 indicates an apparent disparity between the adults based on the type of residence in Indonesia. Someone who lives in an urban area has 1.493 times higher odds than someone in a rural area to utilize outpatient service at the hospital (AOR 1.493; 95% CI 1.489–1.498). Meanwhile, someone who lives in an urban area has 1.075 times higher odds than someone in a rural area to utilize the inpatient facility hospital (AOR 1.075; 95% CI 1.073–1.077). Moreover, someone who lives in an urban area has 1.208 times higher odds than those who live in a rural area to utilize at the same time outpatient and inpatient services at the hospital (AOR 1.208; 95% CI 1.204–1.212).

This analysis indicates that disparities still exist based on the type of residence in Indonesia. Those who live in urban areas have better chances than those who live in rural areas, both in outpatient, inpatient, and both services at the hospital in Indonesia.

In addition to the type of residence, the study found seven control variables to have a significant relationship with hospital utilization. First, the study found age to have a substantial connection with hospital utilization in outpatient, inpatient, and outpatient and inpatient at the same time.

Second, based on gender. Males have a lower odds than females of utilizing outpatient and inpatient services in hospitals. On the other hand, males have a higher odds of using outpatient and inpatient services simultaneously than females.

Third, regarding marital status. Someone who was never in a union has better odds than someone who is divorced/widowed to take advantage of outpatient services but has a lower odds of using other hospital services. On the other side, someone who is married or living with a partner has a higher odds than someone who is divorced/widowed to take advantage of all services at the hospital.

Fourth, according to education level. The analysis results indicate that the better the education level, the better the utilization of services at the hospital. This situation applies to all services in the hospital. Fifth, based on work type, those with all work types have a lower odds than those with other work types to take advantage of hospital services, except for someone who does not work. The study found they those who do not work have a higher odds of using hospital services, both outpatient, inpatient, and outpatient-inpatient, at the same time.

Sixth, the results of the study found that the better the wealth status, the higher the odds of someone using all services in hospitals in Indonesia. Seventh, according to health insurance ownership. Those who have any health insurance have a better odds of taking advantage of the hospital than those who are uninsured. Based on the type of health insurance, the best hospital utilization is those who have both government-run and private-run insurance, then those who have private-run insurance, and finally those who have government-run insurance.

## Discussion

As in most developing countries, urban growth in Indonesia is more advanced than in rural development. As a result, metropolitan regions are particularly appealing to job searchers. Moreover, job seekers, primarily young people, flock to the city searching for work. As a result, the urban population has a younger age than the rural population. The migration of job searchers from rural to urban areas resulted in a higher proportion of unemployed people than in rural areas [18, 28].

The measurements suggest that most rural people work as farmers, confirming Indonesia’s image as an agricultural country. However, because most rural villages still have relatively low levels of education, particularly primary school, the socioeconomics of rural communities are not very favorable. The majority of rural residents are in quintile one or are extremely poor. The condition is the polar opposite of the city’s socioeconomic picture. The increased diversity of labor improves the socioeconomics of urban societies [29, 30].

The study results found that there was still a disparity in hospital utilization based on the type of residence. This condition is related to better health care facilities in urban areas, especially hospitals as referral service facilities [18, 31]. Several previous studies often found that urban areas have better health services. According to the WHO, gender, education, occupation, income, ethnicity, and place of residence are all factors that influence access to health care [32–34].

The study found age to have a significant relationship with hospital utilization in outpatient, inpatient, and outpatient and inpatient simultaneously at the same time. It means that age has a connection with hospital utilization. Age is one of the factors that affect biological organs. Moreover, increasing age accumulates various molecular and cellular damage [35]. Aging is a driving factor for neurodegenerative diseases, cardiovascular diseases, cancer, immune system disorders, musculoskeletal disorders, impaired cognition, mood, and performance [36, 37]. The condition of adults and the aging process causes the utilization of health facilities to increase both in outpatient and inpatient hospitals. Children are different from adults children have a susceptibility to illness. The study in Burkina Faso found that mothers in rural areas often fail to receive care at health facilities for their children due to the sporadic nature of seeking treatment [38]. The findings in this study strengthen the results of the investigation.

Gender was related to hospital utilization. Men are less likely than women to use outpatient and inpatient services. Although on the other hand, men have a higher probability of using outpatient and inpatient services simultaneously than women. A study in India found that almost two-thirds of non-maternity expenditures are for men [39]. Studies in Pakistan mention the dependence of women on male members to get vaccinated and patriarchal decisions to obtain health services [40]. The disparity in hospital utilization occurs because some households allocate more resources to men’s health than women’s. Social service subsidies programs can increase women’s utilization but fail to address gender inequality [39]. A study in the Republic of Serbia stated that women were significantly more likely to use primary health care compared to men. However, the frequency of hospitalization was significantly higher in males than females [41]. This finding indicates that there is still a gender disparity in hospital utilization, both outpatient and inpatient.

On the other hand, the study results indicate that marital status is related to hospital utilization. Someone who is married or living with a partner has a higher odds than someone who is divorced/widowed to take advantage of all the services in the hospital. These results support previous studies showing that married patients have multiple health care utilization at the primary and secondary health care levels [42]. In line with these results, studies in the United States and Puerto Rico reported that married women had a higher tendency to make outpatient visits [43]. Meanwhile, a study in Indonesia stated that divorced, single, and widowed hospitals had better hospital utilization than married [44]. In addition, those who are single and widowed have a higher hospital stay than married [45]. Marital status forms a favorable bond. Marital status also increases the stage of family development [46]. The story of the family stage raises the need to take advantage of health facilities—utilization of these health facilities in general increases along with changes due to marriage.

Based on the level of education, the better the level of education, the better the utilization of hospital services. Education is often associated with knowledge and health awareness [47]. The higher the health knowledge, the higher the attention to hospital services. Studies on rural women in Bangladesh prove that those with higher formal education are more aware of utilizing women’s health services [47]. The results of previous studies reported that the utilization rate of rural community health services was low. The utilization of health services varies by education level, which means the level of education has a positive and significant influence on health services [48]. A study in Congo that examined maternal education levels also found that better education was associated with higher utilization of antenatal care [49]. In line with the survey, a previous study reported that primary education had a lower probability of visiting the hospital than secondary and higher education. On the other hand, those with low education have a more extended stay in hospital than those with higher education [50].

The study results found that type of work was related to hospital utilization. Similar to previous studies, it reported that someone who works has a higher odds than someone who does not work to take advantage of hospital services [44, 51]. Work is related to income and purchasing power, and people who have jobs have sources of revenue, and the existence of payment makes it easier for someone to take advantage of the health services needed [52].

Based on wealth status, the results of the study found that the better the wealth status, the higher the probability of utilizing all hospital services in Indonesia. This study also strengthens the previous finding that the better the level of income/socioeconomic status, the better the utilization of hospital services [19, 53]. Studies in Afghanistan also report that poor women have lower overall institutional delivery rates in public and private facilities [54]. Wealth status significantly contributes to antenatal care utilization in Indonesia and the Philippines [55]. A study in the Philippines reported that increasing health insurance ownership was accompanied by a decrease in inequality in health service utilization [56]. Reflections on the elderly in Vietnam corroborate the finding that a sufficient income is a strong predictor of public health facilities [57]. Maternal health services in Ethiopia are low and uneven and favor more affluent women [58].

Those who have health insurance of all kinds have better odds of taking advantage of the hospital than those who do not have insurance. These results support research on Asian immigrant women. Women who have health insurance are more likely to visit primary care providers and women-only health care providers. Korean women are more likely to see traditional eastern medicine providers than Chinese women [59]. The study in Northern Ghana also found that insurance holdings had an increased chance of taking advantage of both; inpatient and outpatient health services. They reported a history of injury, poor or impoverished health status, and chronic illness [60].

On the other hand, previous studies have found the opposite; the urban poor with insurance are less likely to use hospital services than those without insurance [44]. A study in three Indian states, namely Gujarat, Haryana, and Uttar Pradesh, found no significant relationship between the utilization of inpatient services for those who had insurance and those who did not have insurance [61]. In line with this finding, a study in Vietnam also stated that having health insurance was not a significant predictor of using health facilities [57].

### Strength and limitation

The study analyzes extensive data to depict information on a national scale. On the other hand, the study analyzes secondary data; therefore, the study limits the variables investigated to acceptable variables. Other variables associated with hospital utilization identified in earlier studies cannot be explored, including travel time, travel cost to the hospital, and the type of disease [44, 51, 62]. Furthermore, the study’s limitations prevent it from examining the potential that rural residents are healthier and don’t require hospital care, as evidenced by a prior study that looked at self-reported health (SRH) [63].

## Conclusion

Based on the results, the study concluded that there were urban–rural disparities in hospital utilization in Indonesia. Those who live in urban areas have better odds of using hospitals in Indonesia.

## Data Availability

The data that support the findings of this study are available from the National Institute of Health Research and Development of Indonesia Ministry of Health, but restrictions apply to the availability of these data, which were used under license for the current study, so are not publicly available. However, data are available from the authors upon reasonable request and with permission of the National Institute of Health Research and Development of Indonesia Ministry of Health via the web page http://www.litbang.kemkes.go.id/jasa-permintaan-data-riset.

## Abbreviations

NHI: National Health Insurance
UHC: Universal Health Coverage
SDGs: Sustainable Development Goals
PPS: Probability proportional to size
VIF: Variance inflation factor
AOR: Adjusted odds ratio
CI: Confidence interval
LB: Lower bound
UB: Upper bound
